# Supplementary material for: Surveillance of Antimicrobial Prescriptions in Community Pharmacies Located in Tokyo, Japan
Source: Antibiotics (Basel). 2023 Aug 17;12(8):1325. doi: 10.3390/antibiotics12081325 (PMC10451865; doi:10.3390/antibiotics12081325)
Supplement: Supplementary file 1 [file antibiotics-12-01325-s001.zip › antibiotics-2507119-Supplementary data.pdf]

Table S1. The classifications and formulations of the antimicrobial agents for 42 insurance pharmacies.

Antimicrobial class	Antimicrobial agents and formulations
Penicillin with extended spectrum	ampicillin (capsules), amoxicillin (capsules, fine granules, and tablets), and bacampicillin (tablets)
Combinations of penicillins, incl. beta-lactamase inhibitors	amoxicillin/clavulanic acid (tablets and dry syrup) and sultamicillin (tablets)
1st cephalosporins	cefalexin (tablets, dry syrup, capsules, fine granules, and granules)
2nd cephalosporins	cefuroxime (tablets) and cefaclor (capsules, fine granules, and granules)
3rd cephalosporins	cefixime (capsules and fine granules), cefpodoxime (tablets and dry syrup), cefdinir (tablets, capsules, and fine granules), cefditoren (tablets and fine granules), cefcapene (tablets and fine granules), and cefteram (tablets)
Carbapenems	tebipenem pivoxil (fine granules)
Other cephalosporins and penems	faropenem (tablets and dry syrup)
Fluoroquinolones	ofloxacin (tablets), ciprofloxacin (tablets), norfloxacin (tablets), lomefloxacin (tablets), levofloxacin (tablets and fine granules), moxifloxacin (tablets), prulifloxacin (tablets), garenoxacin (tablets), sitafloxacin (tablets), tosufloxacin (tablets and fine granules), and lascufloxacin (tablets)
Macrolides	erythromycin (tablets, dry syrup, and granules), roxithromycin (tablets), josamycin (syrup), clarithromycin (tablets, dry syrup), and azithromycin (tablets, capsules, and fine granules)
Aminoglycosides	kanamycin (capsules)
Lincosamides	clindamycin (capsules) and lincomycin (capsules)
Tetracyclines	demeclocycline (capsules), doxycycline (tablets), tetracycline (capsules), and minocycline (capsules, tablets, and granules)
Fosfomycin	fosfomycin (tablets and dry syrup)
Anti-tuberculous drug	cycloserine (capsules), rifampicin (capsules), isoniazid (tablets, powder), pyrazinamide (powder), and ethambutol (tablets)
Combinations of sulfonamides and trimethoprim, incl. derivatives	sulfamethoxazole and trimethoprim (tablets and granules)
Combinations for the eradication of *Helicobacter pylori*	lansoprazole/amoxicillin (capsules)/clarithromycin (tablets), rabeprazole/amoxicillin (capsules)/clarithromycin (tablet), vonoprazan/amoxicillin (capsules)/clarithromycin (tablets), lansoprazole/amoxicillin (capsules) and metronidazole (tablets), rabeprazole/amoxicillin (capsules) and metronidazole (tablets), and vonoprazan/amoxicillin (capsules) and metronidazole (tablets)

Other antimicrobials	linezolid (tablets), vancomycin (powder), and rifaximin (tablets)

Table S2. The classifications and formulations of the antimicrobial agents for 12 pharmacies located in front of pediatric clinics.

Antimicrobial class	Antimicrobial agents and formulations
Penicillins with extended spectrum	amoxicillin (fine granules)
Combinations of penicillins, incl. beta-lactamase inhibitors	amoxicillin and clavulanic acid (dry syrup)
1st cephalosporins	cefalexin (dry syrup and fine granules)
3rd cephalosporins	cefixime (fine granules), cefpodoxime (dry syrup), cefdinir (fine granules), cefditoren (fine granules), and cefcapene (fine granules)
Carbapenems	tebipenem pivoxil (fine granules)
Other cephalosporins and penems	faropenem (dry syrup)
Fluoroquinolones	tosufloxacin (fine granules)
Macrolides	erythromycin (dry syrup), josamycin (syrup), clarithromycin (tablets, dry syrup), and azithromycin (fine granules)
Tetracyclines	minocycline (granules)
Fosfomycin	fosfomycin (dry syrup)
Anti-tuberculous drug	cycloserine (capsules), rifampicin (capsules), isoniazid (tablets, powder), pyrazinamide (powder), and ethambutol (tablets)
Combinations of sulfonamides and trimethoprim, incl. derivatives	sulfamethoxazole and trimethoprim (granules)

Table S3. Proportions of oral antimicrobial agents prescribed between April 2013 and 2019 in 12 community pharmacies located in front of pediatric clinics

Antimicrobial class	% of prescriptions each year						
	2013 From April	2014	2015	2016	2017	2018	2019

Penicillins with an extended spectrum	12.8	18.8	22.3	16.0	19.8	36.2	21.9
Combinations of penicillins, incl. beta-lactamase inhibitors	3.3	4.9	12.1	19.7	15.6	9.4	20.4
1st cephalosporins	0.0	0.6	0.6	2.1	5.4	8.0	7.7
3rd cephalosporins	43.6	46.9	38.6	34.7	32.3	27.8	32.8
Carbapenems	1.7	0.4	2.0	2.7	0.8	0.4	0.0
Other cephalosporins and penems	0.5	0.0	2.3	2.0	0.1	0.1	0.0
Fluoroquinolones	10.2	3.8	3.7	5.2	8.7	3.7	3.1
Macrolides	26.0	22.8	17.0	16.2	14.4	11.8	12.8
Tetracyclines	1.4	1.3	0.7	1.0	3.0	1.1	0.0
Fosfomycin	0.4	0.4	0.7	1.0	3.0	1.1	0.0
Anti-tuberculous drug	0.1	0.0	0.0	0.0	0.0	0.0	0.0
Combinations of sulfonamides and trimethoprim, incl. derivatives	0.0	0.0	0.0	0.1	0.0	1.5	1.3
