# Supplementary figures and images for: Surveillance of Antimicrobial Prescriptions in Community Pharmacies Located in Tokyo, Japan
Source: Antibiotics (Basel). 2023 Aug 17;12(8):1325. doi: 10.3390/antibiotics12081325 (PMC10451865; doi:10.3390/antibiotics12081325)

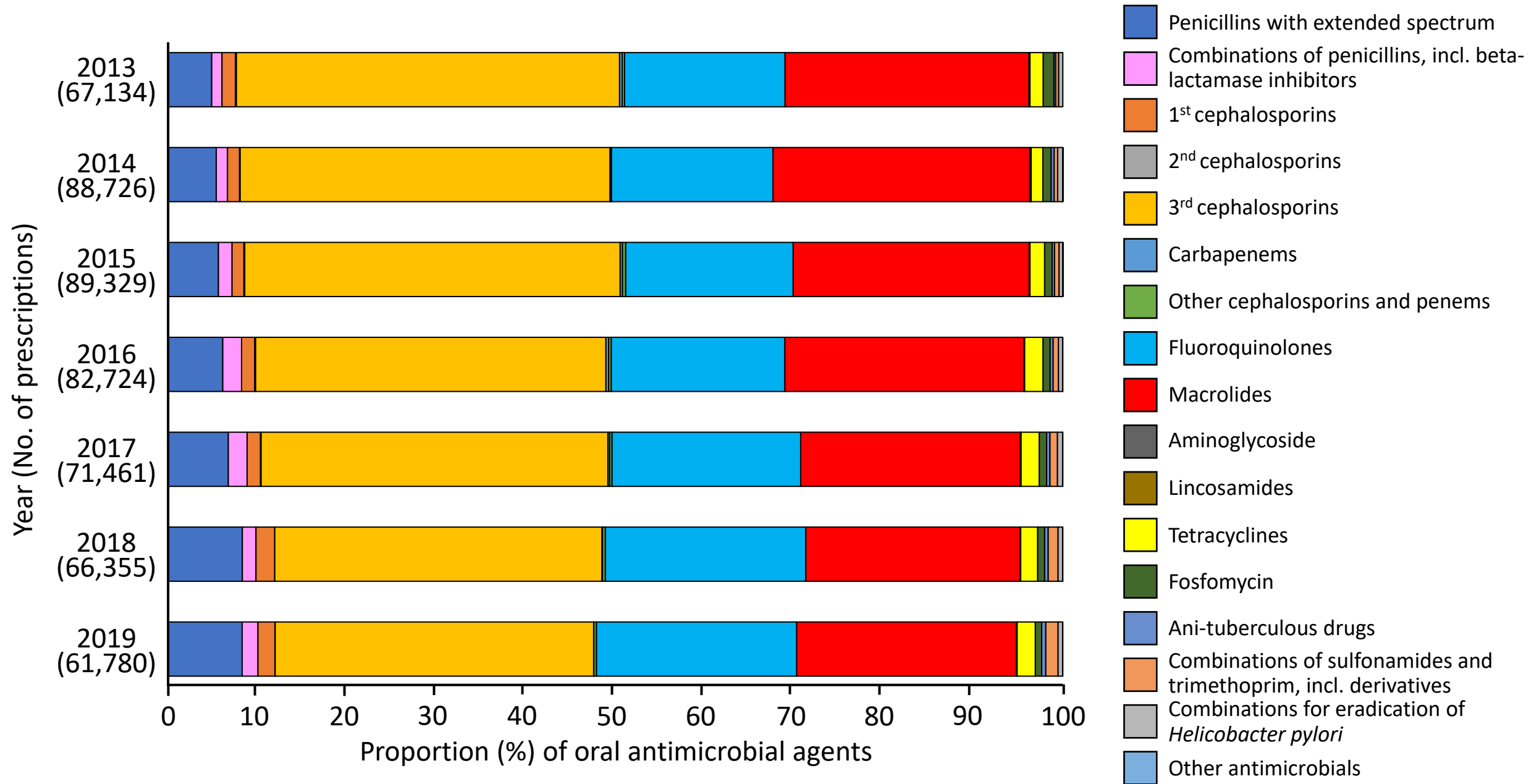

Supplement: Supplementary file 1 [file antibiotics-12-01325-s001.zip › Figure S1.pdf]

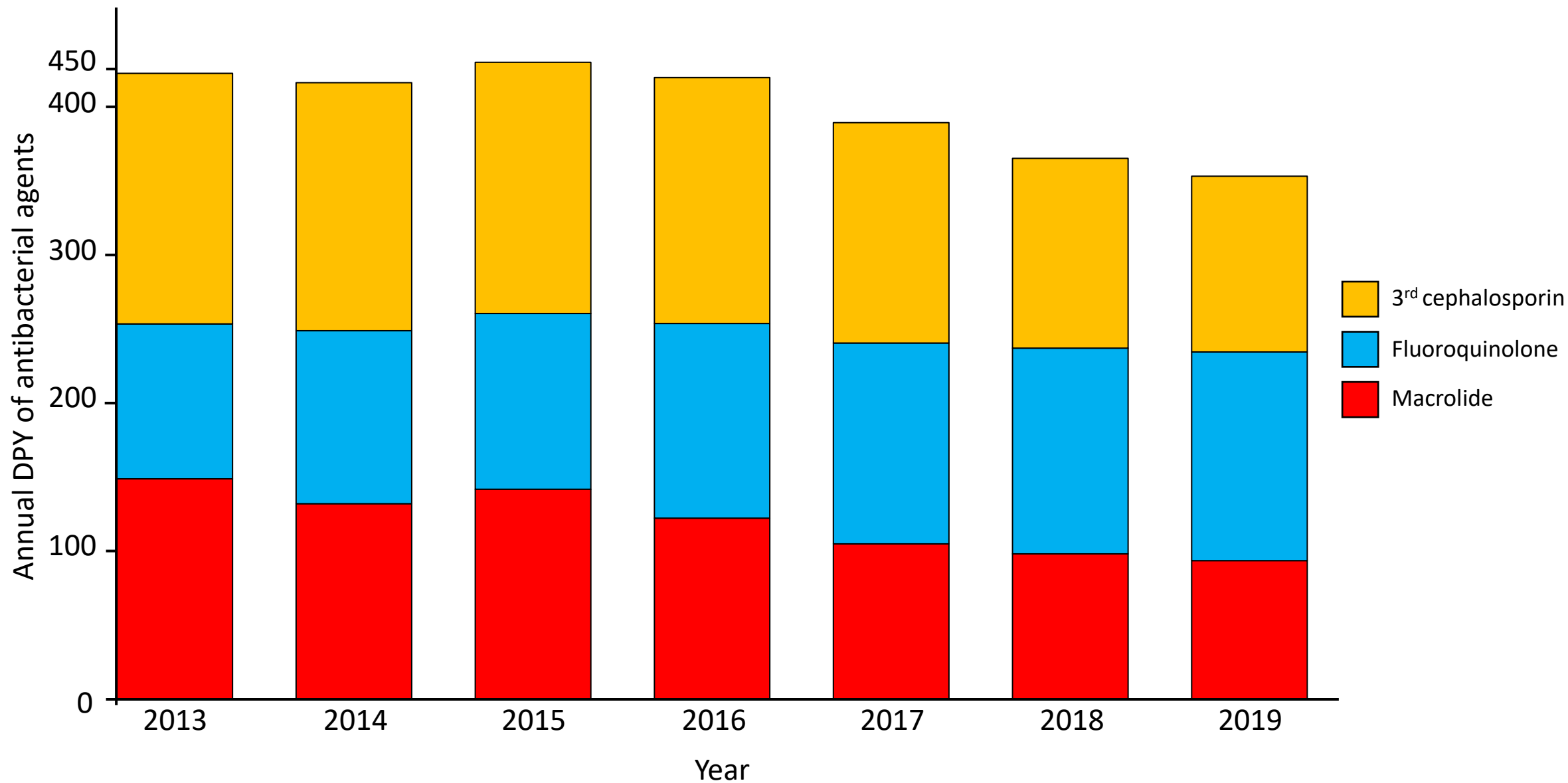

Supplement: Supplementary file 1 [file antibiotics-12-01325-s001.zip › Figure S2.pdf]

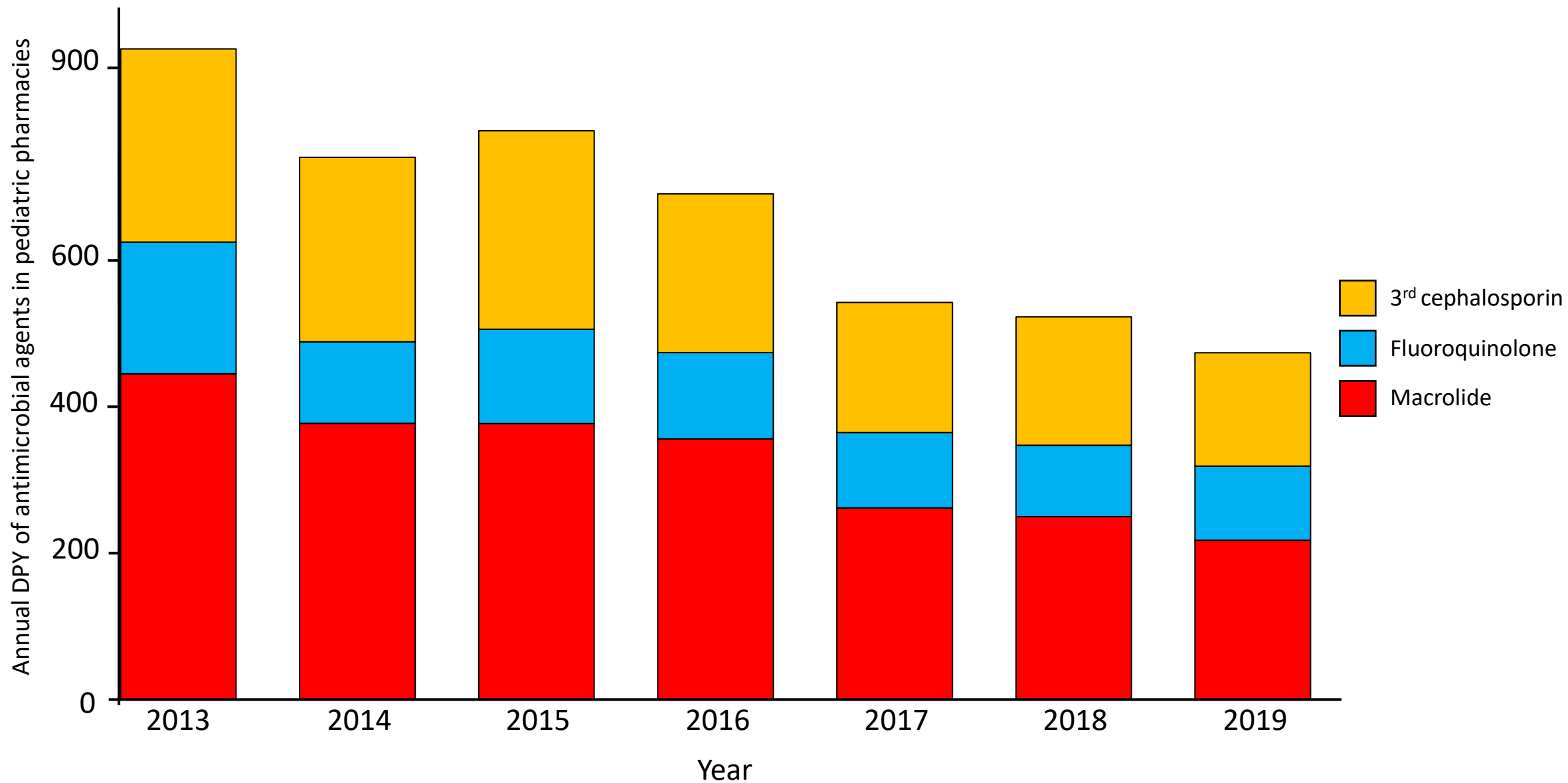

Supplement: Supplementary file 1 [file antibiotics-12-01325-s001.zip › Figure S3.pdf]
